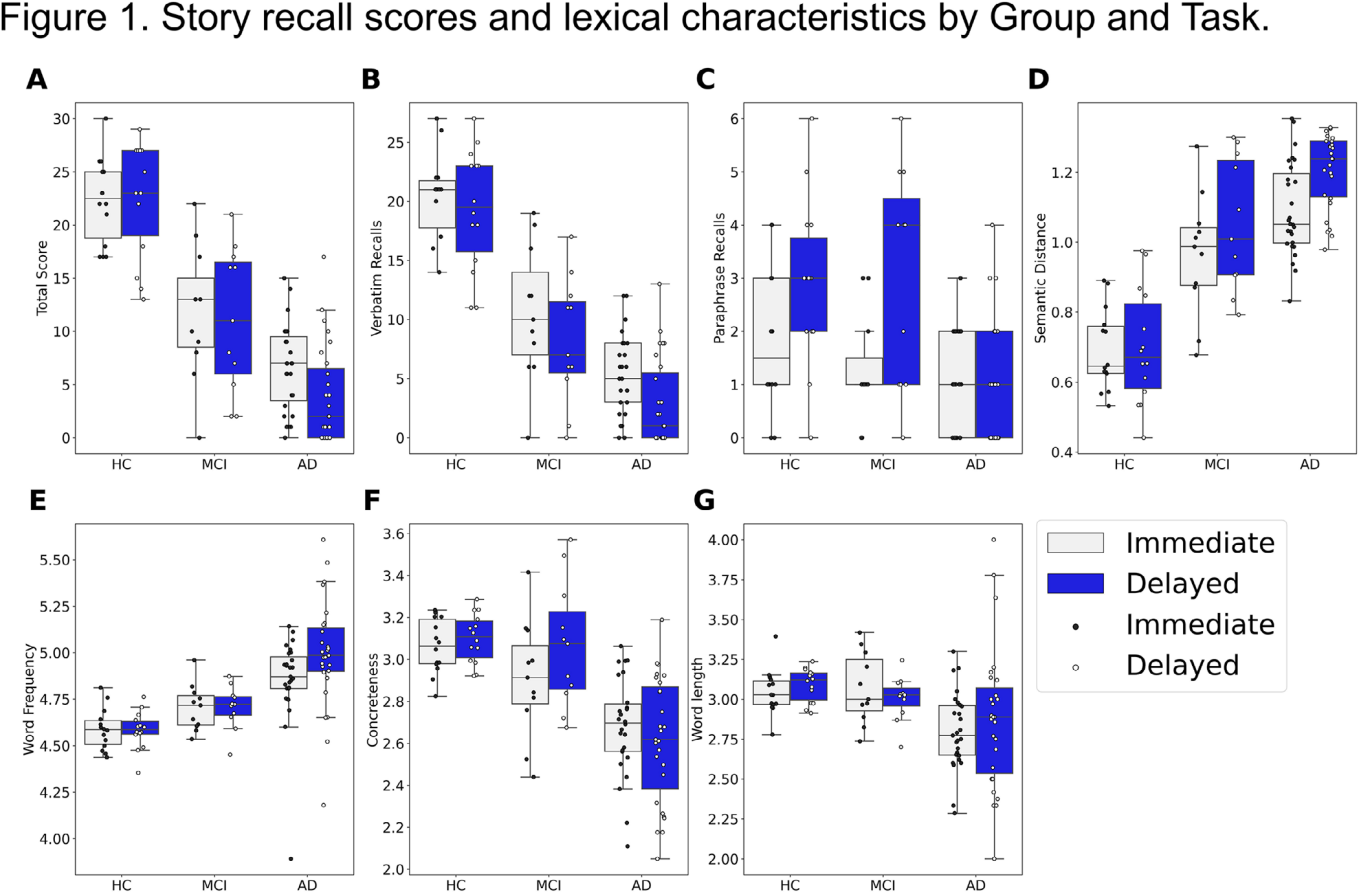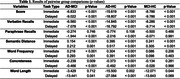# Automated analysis of story recall tasks produced by AD and MCI patients

**DOI:** 10.1002/alz.090515

**Published:** 2025-01-09

**Authors:** Jin‐Seo Kim, Naomi Nevler, David J Irwin, Mark Y Liberman, Sunghye Cho

**Affiliations:** ^1^ Program in Cognitivie Science / University of Pennsylvania, Philadelphia, PA USA; ^2^ Penn FTD Center, University of Pennsylvania, Philadelphia, PA USA; ^3^ Linguistic Data Consortium, University of Pennsylvania, Philadelphia, PA USA

## Abstract

**Background:**

Story recall tasks are often employed in clinical settings to measure verbal episodic memory in individuals with mild cognitive impairment (MCI) and Alzheimer’s disease (AD). Traditional analyses (e.g. total number of recalled words) provide useful but limited information compared to detailed analyses of lexical performance. In this study, we analyzed story recall data produced by patients with AD or MCI using an automated method.

**Method:**

We analyzed digitized recordings of the Craft story recall task produced by 15 individuals with MCI (age=65.8±10.14, females=40%, education=16.07±2.94), 27 individuals with AD (age=65.19±6.82, females=37%, education=15.42±3.01) and 14 healthy age‐matched individuals (HC; age=58.86±16.16, females=50%, education=17.29±2.16). The groups did not differ in any demographic characteristics. Our automated pipeline identified the number of verbatim and paraphrase recalls, calculated semantic similarities between the original story and recalled immediate and delayed stories using word embeddings and rated several lexical characteristics, including word frequency, concreteness, and word length. Two‐way ANOVA tests examined the effect of Group (HC vs. MCI vs. AD), Task (immediate vs. delayed), and their interactions. As the interactions of Group and Task were not significant, post‐hoc analyses examined significant pairwise group differences in immediate and delayed recalls, separately.

**Result:**

Participants with AD produced the fewest verbatim and total recalled words, followed by MCI patients and HC in both immediate and delayed recalls (Figure 1A–B, Table 1). AD patients produced fewer paraphrase recalls compared to the other groups in the delayed recall only (Figure 1C). AD group’s recalled story was also the least similar to the original story, followed by MCI and HC (Figure 1D). Also, individuals with AD produced words that were more frequent and less concrete during immediate and delayed recalls compared to MCI patients and HC (Figure 1E–F). Words produced by AD patients were also shorter than those produced by the MCI and HC groups during the immediate recall (Figure 1G).

**Conclusion:**

Our method highlights that digitized language‐based tests of verbal episodic memory can be automatically analyzed. These capture traditional language features that cannot be extracted manually without massive effort and promise to provide novel, granular, digitized biomarkers that traditional assessment methods of neuropsychological tests cannot show.